# Use of key feature questions in summative assessment of veterinary medicine students

**DOI:** 10.1186/2046-0481-66-3

**Published:** 2013-03-07

**Authors:** Elisabeth Schaper, Andrea Tipold, Jan P Ehlers

**Affiliations:** 1 E-Learning Department, University of Veterinary Medicine Hannover, Foundation, Buenteweg 2, Hannover D-30559, Germany; 2 Clinic for Small Animal Medicine and Surgery, University of Veterinary Medicine Hannover, Foundation, Buenteweg 9, Hannover 30559, Germany

**Keywords:** Key feature questions, Written examination, Reliability, Electronic exam

## Abstract

**Purpose:**

To prove the hypothesis that procedural knowledge might be tested using Key Feature (KF) questions in written exams, the University of Veterinary Medicine Hannover Foundation (TiHo) pioneered this format in summative assessment of veterinary medicine students. Exams in veterinary medicine are either tested orally, practically, in written form or digitally in written form. The only question formats which were previously used in the written e-exams were Type A Single-choice Questions, Image Analysis and Short Answer Questions. E-exams are held at the TiHo using the electronic exam system Q [kju:] by CODIPLAN GmbH.

**Methods:**

In order to examine less factual knowledge and more procedural knowledge and thus the decision-making skills of the students, a new question format was integrated into the exam regulations by the TiHo and some examiner used this for the first time in the computer based assessment. Following a successful pilot phase in formative e-exams for students, KF questions were also introduced in summative exams. A number of multiple choice questions were replaced by KF questions in four computer based assessment in veterinary medicine. The subjects were internal medicine, surgery, reproductive medicine and dairy science.

**Results:**

The integration and linking of KF questions into the computer based assessment system Q [kju:] went without any complications. The new question format was well received both by the students and the teaching staff who formulated the questions.

**Conclusion:**

The hypothesis could be proven that Key Feature questions represent a practicable addition to the existing e-exam question formats for testing procedural knowledge. The number of KF questions will be therefore further increased in examinations in veterinary medicine at the TiHo.

## Background

The University of Veterinary Medicine Hannover Foundation (TiHo) is one of five veterinary educational institutions in Germany. Over 2,400 students, 260 per semester, are enrolled at the TiHo, including PhD students. The 2006 licensure regulations for veterinarians (TAppV) gave veterinary medical educational institutions in Germany more freedom in designing teaching and exams, including the possibility of using new forms of teaching and learning [1].

Each veterinary education institution in Germany has its own exam regulations in which exam requirements and procedures are laid down. Before 2006 most exams were traditionally performed orally. The latest addition to the TAppV allows the use of oral, written and multiple-choice question (MCQ) exams. Due to the alterations in the regulations, written tests in the form of computer based assessment (e-exams) were introduced in the TiHo exam [2].

In general, TiHo uses e-exams for diagnostic, formative and summative assessment [2-5]. Summative e-tests with MCQs for the exam are carried out at TiHo using the computer based assessment system Q [kju:]. This exam system was acquired as a full service, including the Tablet PC, from Codiplan GmbH [2]. Since the introduction of this exam system in April 2008 until May 2012 a total of 159 examinations with 19,294 individual exam “papers” had been carried out. E-exams are now used in 20 subjects of different clinics and institutes at the TiHo (e.g. Small Animal Clinic, Institute of Virology) with a total of 22 exams. In addition, this system is also used for four certificates at the end of the semester in the subjects of chemistry and histology. The question formats which had been used exclusively in these e-exams up until August 2011 were Type A Single-Choice Questions (one-best-answer item format [6]), Image Analysis (e.g. identify a feature on an image such as a fracture or anatomical feature) and Short Answer Questions.

In particular the Type A Single-Choice Question format tests, based on Miller’s knowledge pyramid [7], mainly descriptive knowledge (“knows”) among students, i.e. the knowledge of facts. Well written MCQs can test the second level of the pyramid i.e. application of knowledge and in addition can be ‘case-based’ if designed around a clinical vignette. However, in order to determine the clinical decision-making competence of students, case-based methods are necessary. Therefore a viable solution was needed which would allow case-based e-exams. Different implementations were considered and discussed. These included case-based exams using virtual patients in the form of “long case” exams, simulations, Key Features and adaptive exams, in which questions can be tailored to the individual knowledge of the students. Ultimately the Key Feature question format was chosen [8]. Using Key Feature (KF)-based questions, the decision-making skills of students can be tested in a case-based system. In addition, this format can be integrated into the computer based assessment system Q [kju:].

**Figure 1:**
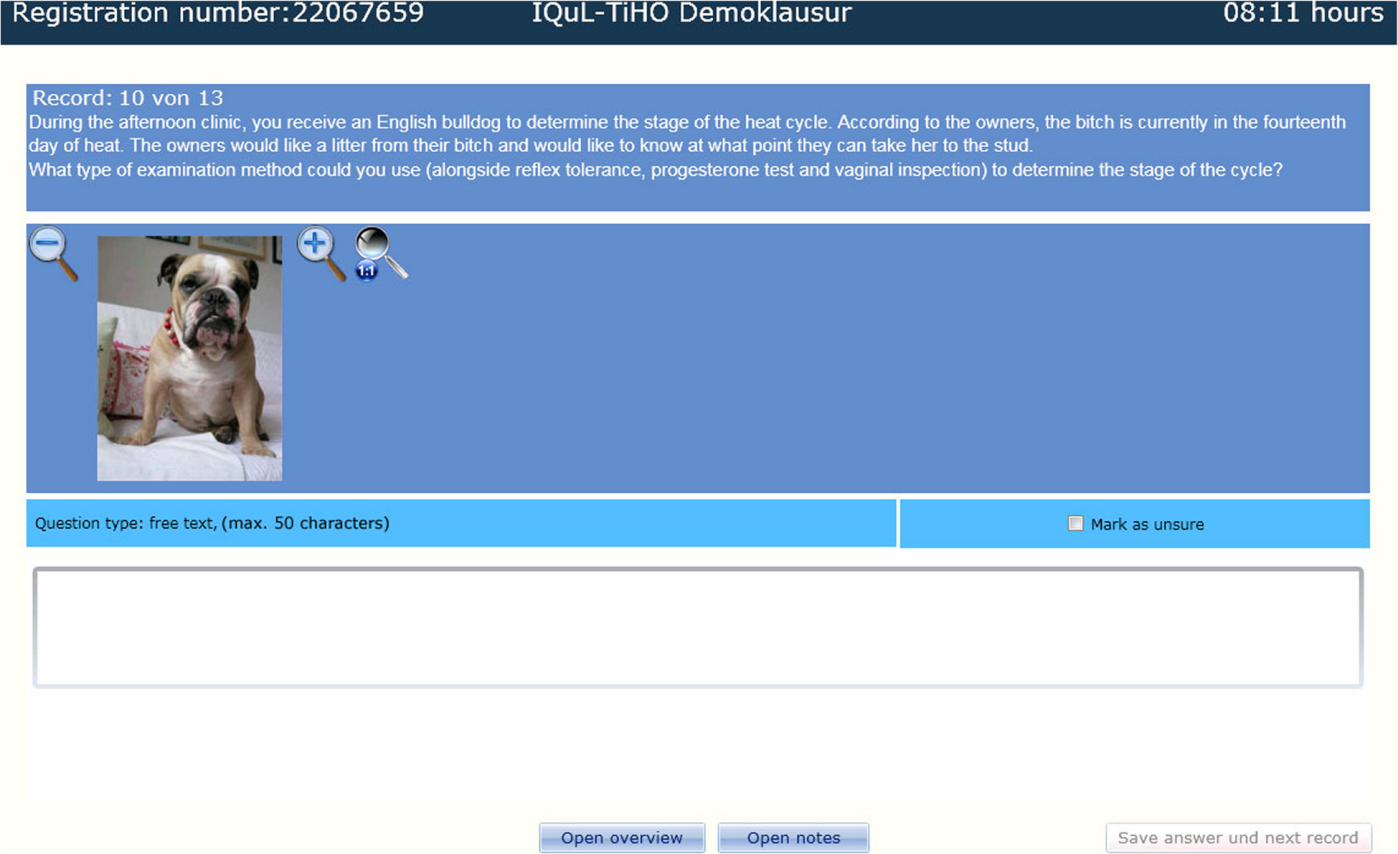
Key Feature question in the demo exam (10), chart 1 of 3, contains the case vignette and a free text question type.

**Figure 2:**
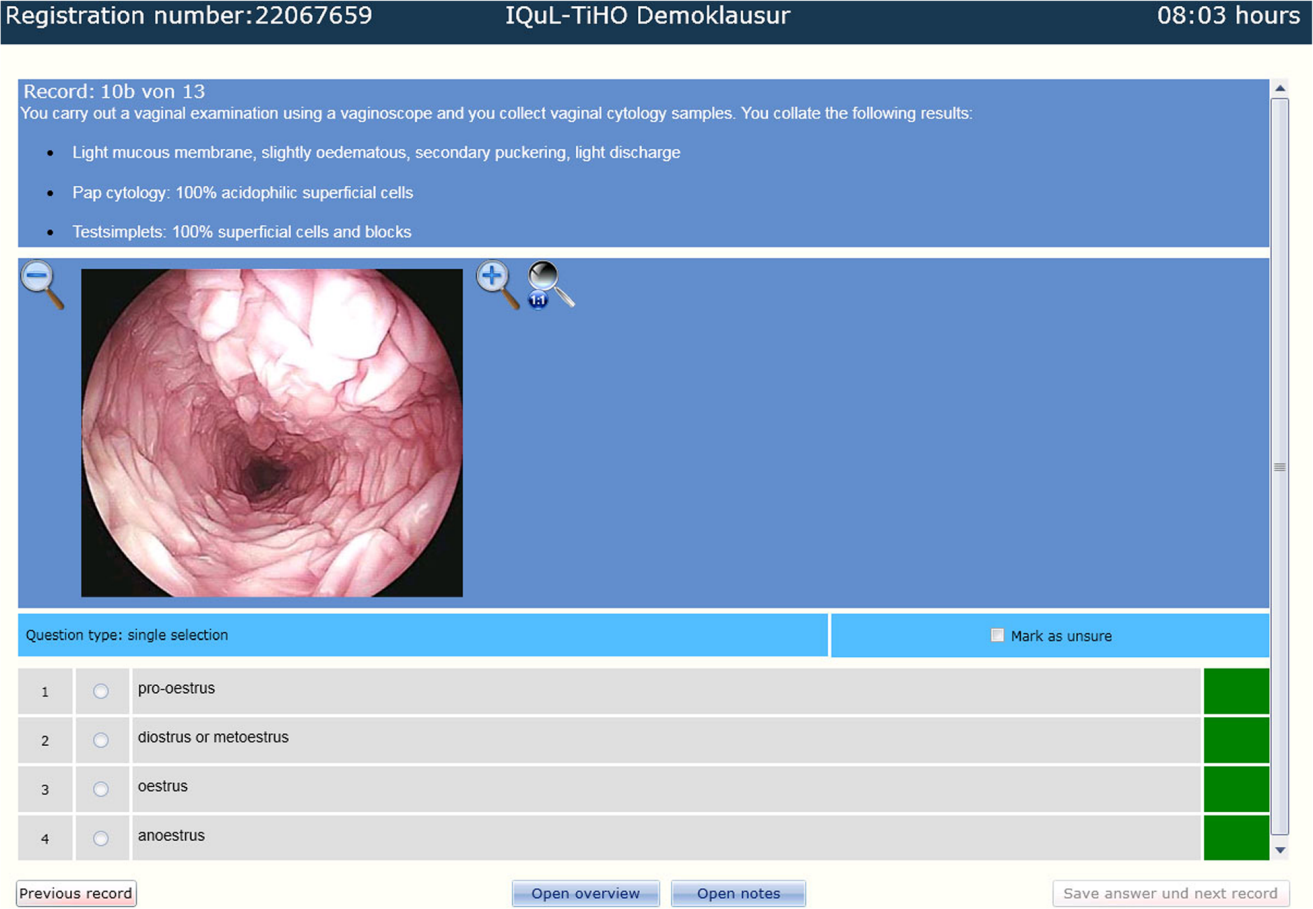
Key Feature question in the demo exam (10b), chart 2 of 3.

**Figure 3:**
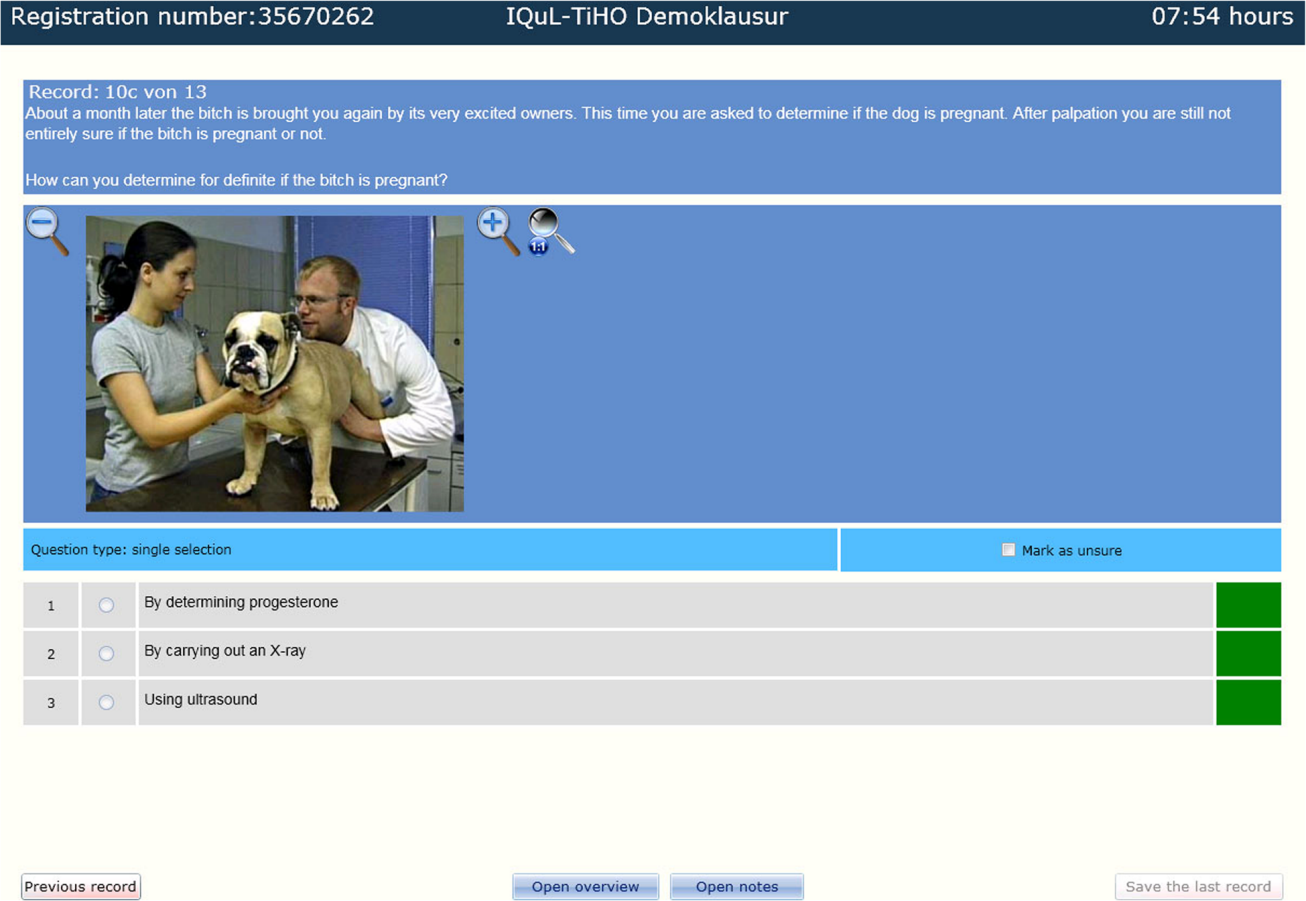
Key Feature question in the demo exam (10c), chart 3 of 3.

**Figure 4:**
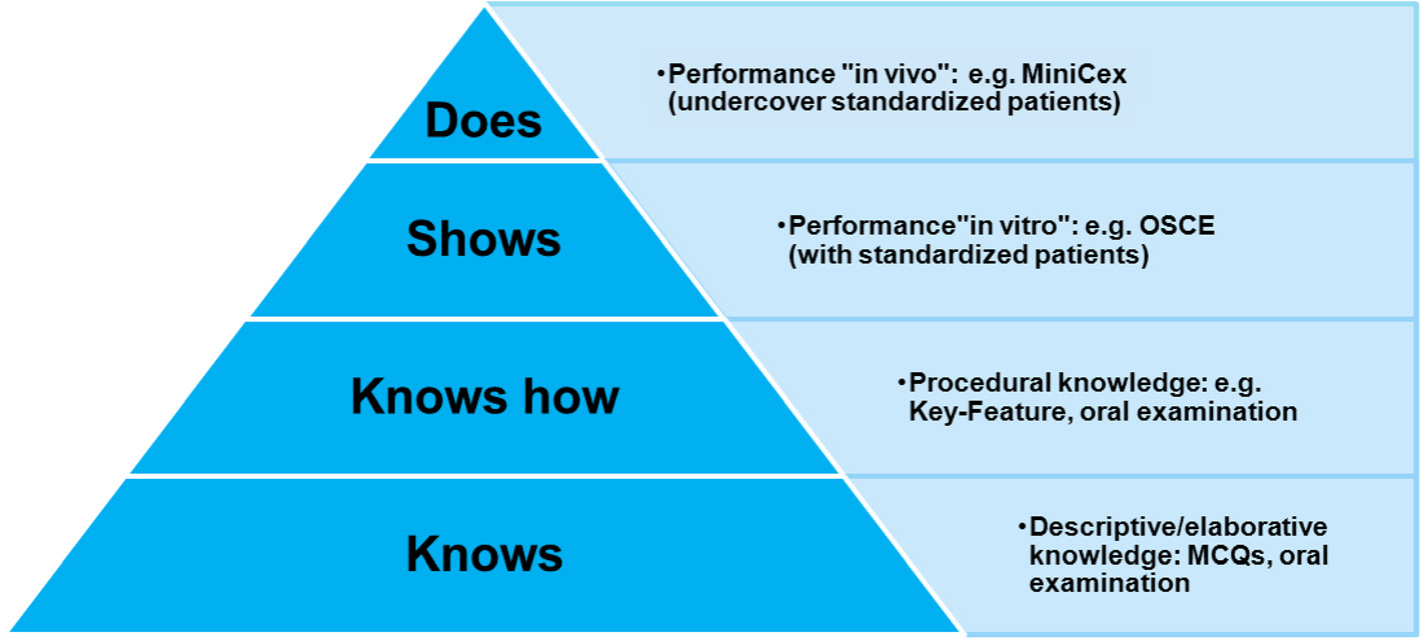
**Miller’s pyramid modified with suitable examination forms according to Kopp et al [**[10]**].** (MiniCEX: Mini Clinical Evaluation Exercise; OSCE: Objective Structured Clinical Examinations; MC: Multiple Choice).

The KF approach was developed by Page and Bordage [9]. KFs were created as a new format for written examinations of clinical decision making (CDM) skills for the Canadian Qualifying Examination in medicine. KFs are defined as critical decisions which must be taken to determine the further course of treatment when dealing with a problem or a patient case [10,11]. In an exam KF can be presented in a concise fashion and used to test the students’ clinical competence extensively. For each clinical case for example, three key questions (e.g. “Which differential diagnoses are plausible?” “Which further medical checks will you instruct?”, “What treatment do you initiate?”) are asked, which will affect the next diagnostic step or treatment significantly (e.g. Figures 1, 2 and 3). The testing of CDM skills by KF problems and the development of KF questions were described by Kopp et al [10]. They characterize the CDM skills as a form of procedural knowledge (“knowledge of how to do things”). This classification was shown by them in the modified Miller’s pyramid [7] (Figure 4).

Although virtual patients are used extensively in teaching [12], so far they or KF questions have been rarely used in summative exams [13].

The aim of this study was to explore the hypothesis that Key Feature questions represent a practicable addition to the existing e-exam question formats for testing procedural knowledge.

## Methods

### Formative exams using Key features

The Key Feature question format was first tested in the spring of 2011 in formative written tests. Formative tests have no impact on a student’s passing or failing. They are a form of learning success control which is not subject to formal assessment.

#### Participants

54 TiHo students who had completed their clinical training year (usually after the 9th to 10th semester) at the clinic for small animals participated in this study. In addition, 11 students from the 6th and 8th semester at the TiHo who had attended a virology elective class also participated and were asked to answer KF questions about the course content.

#### Instruments

The learning and authoring system CASUS® by Instruct AG, Munich [14], was used as a computer based assessment system. The mock exams were held in the computer labs at TiHo. Each participant received personal access credentials to the test system. All responses were centrally recorded and then analysed. Subsequently the advantages and disadvantages of the KF format were discussed with the students.

#### Key feature problems

A KF dealt with one problem or patient case (case vignettes) and always consisted of three consecutive cards with MCQs (Type A – one-best-answer item format and Pick N – this format specifies exactly how many options to select) or Short Answer Questions which had to be answered in sequence. A following sub-question could only be answered, if the previous question had been addressed. The correct answer was displayed in the next follow-up question. It was not possible to navigate back in order to correct a response. Students from the practical clinical years were asked eleven KF questions about small animal medicine, the students from the virology elective course were asked twelve KF questions about the content of the elective course “Viral infections in pigs”.

The KFs were developed by the experts of each clinic after participation in the workshop “Key feature questions: Definition and Process of Creation”. Afterwards a committee of all clinics reviewed all questions. Some additions and changes of the questions regarding specification and understandability of the problem had to be carried out.

### Summative exams using key features

In the summer of 2011, the KF format was first used in the clinical exams. In the written digital parts of the exam, KF questions were used for the subjects internal medicine, surgery and reproductive medicine. In due course, the subject “dairy science” was also integrated into the exams using KF in March 2012 (see Table 1).

**Table 1:** Evaluation of the individual exams of the examination with KFs

**Valuation parameters**	**Internal medicine**	**Surgery**	**Reproductive medicine**	**Dairy science**
Number of participating students	225	242	241	244
Number of KFs in the exam	4 of 60 questions	4 of 60 questions	3 of 60 questions	2 of 60 questions
Average difficulty index of the overall exam	75.76%	73.4%	70.54%	77.36%
Average selectivity of the overall exam	0.29	0.21	0.27	0.29
Cronbach’s α of the overall exam (including KFs)	0.802	0.671	0.776	0.755
Cronbach’s α of the overall exam (excluding KFs)	0.717	0.599	0.761	0.723
Pass grades	96%	96.69%	85.84%	99.59%
KFs correctly answered (average)	87.125%	75.65%	68.87%	83.85%

#### Participants

Between 225 and 244 students of veterinary medicine attended each of the four above-mentioned exams (see Table 1).

#### Instruments

The KF questions were integrated into the computer based assessment system Q [kju:] by CODIPLAN GmbH, Bergisch Gladbach using analogous methodologies to the formative exam questions.

#### Key feature problems

Of the 60 MCQs in the examinations on internal medicine and surgery, four were KFs, three of the 60 questions on reproductive medicine and two of the 60 questions on dairy science. Again, a KF consisted of three consecutive Single Choice Questions.

### Research methods

In order to evaluate the exam results, the difficulty index [15], Cronbach’s α and the selectivity according to “*Pearson’s r”* were calculated. The values of the test performance criteria in the formative exams refer only to the KF questions. In the summative exams, the results refer to the whole exam. Statistical analysis was performed using the software “Itemanalyse ohne SPSS – alles auf einen Streich” (© Dr. H. Stauche, 2013) [16].

To perform an acceptance analysis, the formative exams were followed by focus group discussions. The participants consisted of students from the three courses of the small animal clinic in groups of 20, 18 and 16, 11 students were from the virology elective. The analysis of the focus groups was carried out by two investigators, who independently examined the same transcript of the recorded focus groups interviews. Protocols and results were brought together in consensual discussion without using a software program.

All data from this study was used anonymously and in a confidential way according to the EU Data Protection Directive 95/46/EC. The clearance for this research project was given by the data protection officer of the university. The study was performed under the ethical regulations of the university.

## Results

### Formative exams

A KF question consisted of three sub-questions. Each correctly answered sub-question was awarded a point, as a result of which a maximum of three points per KF question could be achieved. The formative exam with 11 KF questions that was taken by 54 students of the clinical practice year resulted in a Cronbach’s α of 0.585, an average difficulty index of 77.39% and an average selectivity of 0.26. The 11 students of the virology elective were asked 12 KFs. The analysis returned a Cronbach’s α of 0.761, an average difficulty index of 70.25% and an average selectivity of 0.34. Due to the small number of 11 participants, these results have a low informative value.

The acceptance of the KF format was very high amongst the students. During the focus group discussions, they explained that the KF format allowed them to stay within a subject area for longer. The students also judged the relevance of the KF questions to be high. The focus groups also fiercely debated whether it was not possible to increase the competence-based element of oral examinations.

### Summative exams

A KF question also consisted of three sub-questions. One point was awarded per KF question if at least two sub-questions were answered correctly. If no or only one sub-question was answered correctly, the candidate received no points for this question. The results are shown in Table 1 and relate to each exam as a whole. The average difficulty index of these four exams was between 70.54% and 77.36%, the average separation efficiency between 0.21 and 0.29 and Cronbach’s α of the overall exam including KFs between 0.671 and 0.802 and without KFs between 0.599 and 0,761. Overall, between 85.84% and 99.59% of the students passed their exams. Of the KF questions on internal medicine 87.125% were answered correctly by the students, of those on surgery 75.65%, of those on reproductive medicine 68.87% and of those on dairy science 83.85%.

The integration and linking of KF questions into the computer based assessment system Q [kju:] worked successfully. Both the students and the faculty gave good feedback on this case-based approach.

## Discussion

The TiHo changed their exam system from only oral exams to a mixture of written (most electronic) and practical exams (LIT). With educational research the TiHo tries to find and establish more reliable and valid test systems and to carry out exams in a more competence based manner. The TiHo has currently reached the point where it uses computer based assessment (Single Choice Questions, Image Analysis and Key Features) for assessing students of veterinary medicine in addition to traditional exam methods (practical, oral and written tests). The aim is not only to test descriptive but also procedural knowledge in written computer based assessment [9].

Huwendiek *et al*[17]. have already used KFs in a computer and case-based exam in a study involving students of human medicine using long selection lists (“long menus”) to investigate the feasibility, acceptance and test statistical quality and came to the conclusion that all of these three aspects are achieved and that therefore the KF approach with long selection lists is suitable for computer-based testing of applied knowledge. Rotthoff *et al.* also report on the use of KFs with Long Menu Questions (LMQs) [18]. Fischer *et al*[19] conducted a study to develop and validate a KF-based exam for medical students. Using 15 KFs, they achieved a reliability of 0.65 (Cronbach’s α) and extrapolated that 25 KFs are needed in an exam in order to achieve a Cronbach’s α of 0.75. With respect to the test quality criteria they found positive results. Both Farmer and Page [20] and Hatala and Norman [21] in principle also came to a positive evaluation of the KF format.

In principle the idea is not to change the computer based assessment at the TiHo completely but to use KFs as a useful complement to the old question formats of MC exams. Until now, thirteen KF questions in total have been used in four different exam subjects. There were no practical problems. The acceptance of the new question format is high both amongst the faculty and the students, so the plan is to introduce this question format into more exams in the future. In the long term, the ratio in exams should also shift in favour of KFs. Compared to conventional Single-Choice Questions, the design of KF questions may be more complex. Kopp and Moeltner [10] therefore propose a national database of KF questions for human medicine for example in order to make a pool of high-quality KF questions available. Because many exam regulations do not cite KF as an acceptable question format, it is generally relatively unknown. To strengthen the use of KFs in exams and thereby also to provide assistance, TiHo has presented this format in various training programs for teaching staff and at inter-university (KELDAT, http://www.keldat.org) and interdisciplinary collaborations (N^2^E^2^, http://www.n2e2.de).

The reliability coefficient of summative tests should ideally exceed 0.8 [22]. In the summative exams conducted to date, this value was reached or almost reached with Cronbach’s α. This value could in principle be improved in formative exams by increasing the number of items [19]. It will also be necessary to consider how the KFs alter the valuation parameters (Table 1). Due to the low proportion of KF questions in the exams, there is no data yet on the difficulty index, the selectivity and Cronbach’s α regarding the KF questions. This will be carried out when a sufficient proportion of KFs has been reached. We have already noted that the reliability of each overall exam did not deteriorate through the use of KFs when compared to the previous year’s results. The Cronbach’s alpha of the overall examination with KF items included is slightly higher than when these items are removed. However the relevance of this finding is questionable since other research has shown that the correlation between MCQ type exams and Key Feature exams is only moderate at best. KFs are ultimately used to improve the validity while retaining reliability [2]. It is currently being reviewed whether the use of KF questions in exams has an impact on the design of other MCQs: e.g. writing the vignettes in a case-based way and testing procedural knowledge instead of simply requiring students to remember isolated facts. Furthermore, the assessment scheme of the formative exam will be carried over in the upcoming trials. Each KF question consists of three sub-questions. Each correctly answered question will now be worth one point so that for each KF question in an exam, a total of three points can be achieved.

## Conclusions

In summary we can say that the first use of KFs in formative and summative exams was very successful and that the hypothesis could be proven that Key Feature questions represent a practicable addition to the existing electronic written exams. Both the integration of the KFs into the computer based assessment system Q [kju:] and acceptance by the faculty and students was positive. Nevertheless, before it can be routinely used in exams there is still some work to be done. With the coming exams and the accompanying increase in the share of KF questions, test data such as the difficulty index, selectivity and Cronbach’s α will be collected, presented and discussed.

## Competing interests

The authors declare that they have no competing interests.

## Authors’ contributions

ES and JE developed the idea and the study design and collected, analyzed and interpreted the data. ES was the primary author of the paper. JE and AT acted as supervisors of the work. All authors read and approved the final manuscript.
